# Closed testing using surrogate hypotheses with restricted alternatives

**DOI:** 10.1371/journal.pone.0219520

**Published:** 2019-07-12

**Authors:** John M. Lachin, Ionut Bebu, Michael D. Larsen, Naji Younes

**Affiliations:** 1 The Biostatistics Center, Department of Biostatistics and Bioinformatics, Milken Institute School of Public Health, George Washington University, Rockville, Maryland, United States of America; 2 St. Michael’s College, Colchester, Vermont, United States of America; Indiana University Bloomington, UNITED STATES

## Abstract

**Introduction:**

The closed testing principle provides strong control of the type I error probabilities of tests of a set of hypotheses that are closed under intersection such that a given hypothesis *H* can only be tested and rejected at level *α* if all intersection hypotheses containing that hypothesis are also tested and rejected at level *α*. For the higher order hypotheses, multivariate tests (> 1*df*) are generally employed. However, such tests are directed to an omnibus alternative hypothesis of a difference in any direction for any component that may be less meaningful than a test directed against a restricted alternative hypothesis of interest.

**Methods:**

Herein we describe applications of this principle using an *α*-level test of a surrogate hypothesis $\tilde{H}$ such that the type I error probability is preserved if $H\Rightarrow \tilde{H}$ such that rejection of $\tilde{H}$ implies rejection of *H*. Applications include the analysis of multiple event times in a Wei-Lachin test against a one-directional alternative, a test of the treatment group difference in the means of *K* repeated measures using a 1 *df* test of the difference in the longitudinal LSMEANS, and analyses within subgroups when a test of treatment by subgroup interaction is significant. In such cases the successive higher order surrogate tests can be aimed at detecting parameter values that fall within a more desirable restricted subspace of the global alternative hypothesis parameter space.

**Conclusion:**

Closed testing using *α*-level tests of surrogate hypotheses will protect the type I error probability and detect specific alternatives of interest, as opposed to the global alternative hypothesis of any difference in any direction.

## Introduction

The closed testing principle of Marcus, Peritz and Gabriel [1] provides strong control of the type I error probability, the so-called family-wise error rate (FWER), over a set of tests of multiple hypotheses. The basic principle is that a given elemental null hypothesis can be tested and rejected at level *α* if all higher order intersection hypotheses containing it have also been tested and rejected at level *α*. In this case the type 1 error probability for the set of hypotheses, both elemental (i.e. simple) and joint (i.e. intersections), will be protected at level *α* provided that each hypothesis is tested using an *α*-level test, meaning that the type 1 error probability associated with a given test of a given hypothesis is no greater than *α*, multiple testing aside. Hsu [2] describes various applications. Henning and Westfall [3] provide a review of historical and recent developments.

The most common application of closed testing is pairwise tests of group differences in a multiple *K* > 2 group trial in which we wish to test the equality of the *K* groups by conducting *K*(*K* − 1)/2 pairwise comparisons with strong control of the type I error probability for the set of tests. Let *μ*_*j*_ denote the expected value of the outcome (mean, proportion, etc.) for the *j*th group 1 ≤ *j* ≤ *K*. Consider the case of *K* = 4 groups with 6 pairwise tests. In this case we start with a test of the joint null hypothesis *H*_0,1234_: *μ*_1_ = *μ*_2_ = *μ*_3_ = *μ*_4_ (the highest order interaction hypothesis) against the alternative *H*_1,1234_: *μ*_*j*_ ≠ *μ*_*k*_ for at least one pair of groups among 1 ≤ *j* < *k* ≤ *K* = 4.

Closed testing can also be applied to tests of the difference between two groups for multiple outcomes. Let *θ*_*j*_ refer to the difference between the two groups for the *j*th outcome and assume that we wished to test the individual hypotheses *H*_0,*j*_: *θ*_*j*_ = 0, *j* = 1, …, *K*, with control of the type I error probability for the set of *K* tests. Consider a test of the hypothesis *H*_0,1_: *θ*_1_ = 0. This hypothesis can be rejected at level *α* if it and all intersection hypotheses containing it are also rejected at level *α*. This entails testing the set of hypotheses presented in Table 1 starting with the *K*-level intersection hypothesis. This is a simple testing tree.

**Table 1:** Intersection hypotheses containing *H*_0,1_: *θ*_1_ = 0 in the context of testing the joint null hypothesis of no difference between groups in the means of *K* = 4 outcome measures. Also shown are the equivalent hypotheses in terms of joint tests of specific mean values. In order to reject *H*_0,1_ at level *α*, all of these hypotheses must be nominally statistically significant at level *α*.

	Intersection Hypotheses	Equivalent Hypothesis
Order 4:	*H*_0,1_ ∩ *H*_0,2_ ∩ *H*_0,3_ ∩ *H*_0,4_	*H*_0,1234_: *θ*_1_ = *θ*_2_ = *θ*_3_ = *θ*_4_ = 0
Order 3:	*H*_0,1_ ∩ *H*_0,2_ ∩ *H*_0,3_	*H*_0,123_: *θ*_1_ = *θ*_2_ = *θ*_3_ = 0
	*H*_0,1_ ∩ *H*_0,2_ ∩ *H*_0,4_	*H*_0,124_: *θ*_1_ = *θ*_2_ = *θ*_4_ = 0
	*H*_0,1_ ∩ *H*_0,3_ ∩ *H*_0,4_	*H*_0,134_: *θ*_1_ = *θ*_3_ = *θ*_4_ = 0
Order 2:	*H*_0,1_ ∩ *H*_0,2_;	*H*_0,12_: *θ*_1_ = *θ*_2_ = 0
	*H*_0,1_ ∩ *H*_0,3_;	*H*_0,13_: *θ*_1_ = *θ*_3_ = 0
	*H*_0,1_ ∩ *H*_0,4_;	*H*_0,14_: *θ*_1_ = *θ*_4_ = 0
Order 1:	*H*_0,1_: *θ*_1_ = 0	*H*_0,1_: *θ*_1_ = 0

For *K* = 4 outcomes, the parameter estimates $\hat{\theta }={\left[{\hat{\theta }}_{1}\;{\hat{\theta }}_{2}\;{\hat{\theta }}_{3}\;{\hat{\theta }}_{4}\right]}^{T}$ are jointly asymptotically normally distributed with expectation ***θ*** and a consistently estimable covariance matrix **Σ**. Then the order 4 hypothesis *H*_0,1234_: ***θ*** = **0** could be tested using a *T*^2^-like test of the form

$$\begin{array}{c} X_{1234}^{2}={\hat{\theta }}^{\prime }{\hat{\Sigma }}^{-1}\hat{\theta } \end{array}$$ (1)

that is asymptotically distributed under *H*_0,1234_ as chi-square on 4 *df*. Then an order 3 joint null hypothesis, such as *H*_0,123_, could be tested using a *T*^2^-like test of the form

$$\begin{array}{c} X_{123}^{2}={\hat{\theta }}^{\prime }C{\left(C^{\prime }\hat{\Sigma }C\right)}^{-1}C^{\prime }\hat{\theta } \end{array}$$ (2)

using a matrix such as

$$\begin{array}{c} C^{\prime }=[\begin{array}{cccc} 1 & 0 & 0 & 0\\ 0 & 1 & 0 & 0\\ 0 & 0 & 1 & 0 \end{array}] \end{array}$$ (3)

that is asymptotically distributed as chi-square on 3 *df* under the joint null hypothesis *H*_0,123_. Similar tests can be applied to each order 2 hypothesis. Then the elementary hypotheses, such as *H*_0,1_, could be tested using a simple *t*- or *Z*-test. Thus, *H*_0,1_ would be rejected if the tests of *H*_0,1234_, *H*_0,123_, *H*_0,124_, *H*_0,134_, *H*_0,12_, *H*_0,13_, *H*_0,14_ and *H*_0,1_ were all nominally significant at level *α*. A similar table of hypotheses and rejection criteria would apply to the closed testing for the other 3 elementary hypotheses *H*_0,2_, *H*_0,3_, and *H*_0,4_.

In addition to the hierarchy of *T*^2^-like tests as above, Lehmacher, Wassmer and Reitmeir [4] also describe application to other tests of the differences between means for multiple quantitative outcomes, such as the O’Brien [5] Ordinary Least Squares (OLS)-based test based on the sum of the mean differences over the set of *K* measures. These and other *α*-level tests are also shown to provide strong control of the type I error probability. Wassmer et al. [6] also provide an overview of procedures for analysis of multiple, principally quantitative, outcomes that contrasts omnibus versus directional alternatives.

More generally, consider that we wish to test a set of *K* null hypotheses H closed under intersection, i.e. if H,K∈H, then H∩K∈H as well. Let $H_{T}\in H$ denote the subset of true null hypotheses, $H_{T}$, where rejecting any hypothesis in $H_{T}$ is a type I error. Then let $H_{T}^{⋆}$ be the intersection of all true elemental hypotheses in $H_{T}$, i.e. the highest order true null intersection hypothesis to be tested, where the rejection region ensures that $\mathrm{Pr}(\text{reject}\;H_{T}^{⋆})\leq \alpha$. Also, let *H* be another true null hypothesis $H\in H_{T}$ where $H\neq H_{T}^{⋆}$. Then the order constraint above ensures that $H_{T}^{⋆}$ will precede *H* in the testing order. Since the testing of *H* is conditional on the rejection of $H_{T}^{⋆}$, then

$$\begin{array}{c} \mathrm{Pr}\left(\text{reject}\;H\right)=\mathrm{Pr}\left(\text{reject}\;H\cap \;\text{reject}\;H_{T}^{⋆}\right)\leq \mathrm{Pr}\left(\text{reject}\;H_{T}^{⋆}\right)\leq \alpha . \end{array}$$ (4)

Since $H_{T}^{⋆}$ is always the first true null to be tested, and since $\mathrm{Pr}(\text{reject}\;H_{T}^{⋆})\leq \alpha$, the cumulative probability of all further type I errors cannot exceed *α*.

Closed testing typically employs an efficient (e.g. UMP) test of each null hypothesis against a global alternative hypothesis such as the *T*^2^-like test *H*_0,1234_: ***θ*** = **0** of joint equality against the alternative *H*_1,1234_: ***θ*** ≠ **0** that the group difference for at least one of the outcomes is unequal to zero. However, from (4), the only requirement for closed testing to control the family-wise error rate at the desired level *α* is that each test employed be an *α*-level test [3], meaning that the type I error probability of a test does not exceed the desired level *α* under that null hypothesis. Thus, closed testing can also be applied using a test directed towards a restricted alternative hypothesis, such as the one-directional or one-sided alternative hypothesis *H*_1,1234_: ***θ*** > **0** where positive values of ***θ*** are considered beneficial. In this case the test is directed to a restricted alternative hypothesis that represents a region of the parameter space of greater interest than would be provided by the usual multiple *df* omnibus test of *H*_0_.

More generally, closed testing can also be employed using a *surrogate test* of a *surrogate hypothesis*. Let *H* be a null hypothesis of interest. We will say that a hypothesis $\tilde{H}$ is a surrogate hypothesis for *H* if it satisfies

$$\begin{array}{c} H\Rightarrow \tilde{H} \end{array}$$ (5)

where rejection of $\tilde{H}$ implies rejection of *H*. For example, consider a test of *H*_0,12_: *θ*_1_ = *θ*_2_ = 0 in Table 1 against the alternative *H*_1,12_: *θ*_1_ ≠ 0 and/or *θ*_2_ ≠ 0. A surrogate test could be conducted using ${\tilde{H}}_{0,12}:\theta_{1}=\theta_{2}$ against the alternative ${\tilde{H}}_{1,12}:\theta_{1}\neq \theta_{2}$. Clearly $H_{0,12}\Rightarrow {\tilde{H}}_{0,12}$ and rejection of ${\tilde{H}}_{0,12}$ implies rejection of *H*_0,12_. Even though the efficiency of the test of $\tilde{H}$ may differ from that of the usual test of *H*, $\tilde{H}$ is still is an *α*-level test and this testing strategy preserves the type I error probability at ≤ *α* for the set of tests closed under intersection.

We now present specific applications, starting with the analysis of multiple event-time outcomes (e.g. MACE in a cardiovascular trial) following a one-directional Wei-Lachin multivariate test of a combination of outcomes, with a computational example. This is followed by a description of tests of treatment group differences in means of *K* repeated measures over time where the tests of intersection hypotheses are conducted using tests of the longitudinal LSMEANS rather than *T*^2^-like MANOVA omnibus tests. We then describe testing the treatment difference between two groups within multiple subgroups following a test of treatment by subgroup interaction (i.e. homogeneity). This is accompanied by the computation of the operating characteristics of the traditional closed testing and the surrogate closed testing for this application.

## Components of the MACE composite outcome

We first apply closed testing using surrogate hypotheses to the assessment of the significance of treatment group differences for elements of a composite time-to-event outcome such as a Major Adverse Cardiovascular Event (MACE) using the times to one or more of a set of possible component events such as cardiovascular (CV) death, non-fatal myocardial infarction (MI), non-fatal stroke or non-fatal congestive heart failure, so called 4-point MACE. Herein we compare traditional closed testing using *T*^2^-like “MANOVA” omnibus tests on multiple *df* to surrogate closed testing using Wei-Lachin [7] 1 *df* tests against one-directional restricted alternatives, and also to the commonly used time-to-first-event analysis.

Let *β*_*j*_ denote the log hazard ratio for treatment versus control for a Cox PH model analysis of the time to the *j*th of *K* different types of events including multiple types for a given patient, e.g. time to the first non-fatal MI and time to CV death for a patient who experiences both types of event. The *K* separate models generate a vector of coefficient estimates $\hat{\beta }=({\hat{\beta }}_{1}\ldots \;{\hat{\beta }}_{K}{)}^{T}$ that is asymptotically normally distributed with expectation ***β*** = (*β*_1_… *β*_*K*_)^*T*^ and with a covariance matrix **Σ** with elements

$$\begin{array}{ccc} \sigma_{j}^{2} & = & V({\hat{\beta }}_{j})\text{,}\;j=1,\ldots ,K\\ \sigma_{jk} & = & Cov({\hat{\beta }}_{j},{\hat{\beta }}_{k})\text{,}\;1\leq j<k\leq K. \end{array}$$ (6)

Estimates of the covariances $\left\{{\hat{\sigma }}_{jk}\right\}$ can be provided by partitioning the model-based information sandwich as described in Lachin and Bebu [7], or using the method of Wei, Lin and Weissfeld [8] that employs the Lin and Wei [9] estimate of the observed information that is robust to departures from the proportional hazards assumption. Both approaches may also be adjusted for other covariates, and provide the estimate of the joint covariance matrix $\hat{\Sigma }$ of the treatment group coefficients.

Typically, traditional closed testing of the group differences for the *K* outcomes would start with a test of the global *K*-order null hypothesis versus the global or omnibus alternative hypotheses:

$$\begin{array}{c} H_{0}\text{:}\;\;\beta =0\;versus\;H_{1O}\text{:}\;\;\beta \neq 0 \end{array}$$ (7)

that tests for any difference or combination of differences between groups in any direction, such as where the treatment is beneficial for some outcomes but harmful for others. Using a consistent estimate $\hat{\Sigma }$, the *T*^2^-like Wald test of *H*_0_ versus the global alternative *H*_1*O*_ is provided by

$$\begin{array}{c} X_{O}^{2}={\hat{\beta }}^{\prime }{\hat{\Sigma }}^{-1}\hat{\beta } \end{array}$$ (8)

that is asymptotically distributed as chi-square on *K* *df*. If this *K*-order test is significant at level *α*, then one can continue to conduct the *K* − 1 order tests, etc. The traditional closed testing structure would entail tests of the set of hypotheses presented in Table 1.

Alternately, surrogate closed testing of such a multivariate or composite outcome could be conducted using a test that is directed to a one-directional alternative hypothesis. Assume that *β*_*j*_ < 0 represents a beneficial effect of treatment for the *j*th outcome. For the *K*-order test the one-directional alternative hypothesis specifies that

$$\begin{array}{c} H_{1<}\text{:}\;\{\left(\beta_{1}\leq 0\right)\cap \left(\beta_{2}\leq 0\right)\cap \ldots \cap \;\left(\beta_{K}\leq 0\right)\}\;\text{and}\;\sum_{j=1}^{K}\beta_{j}<0. \end{array}$$ (9)

This surrogate hypothesis specifies that the experimental treatment has a beneficial or neutral effect on each component event (*β*_*j*_ ≤ 0) and is superior for one or more outcomes $\left(\sum_{j=1}^{K}\beta_{j}<0\right)$. Thus, this restricted alternative hypothesis is directed to regions in the *K*-dimensional parameter space where there is a preponderance of benefit for the set of *K* outcomes, though not necessarily to the same degree, with no overt harm for any outcome.

Recently, Lachin and Bebu [7] described the application of the 1 *df* Wei-Lachin robust one-directional test to such data. The test is based on the simple sum, or equivalently the unweighted mean, of the Cox PH model coefficients, or log hazard ratios representing the treatment group difference for each component event, where different types of events in the same subject are included in the analysis of the different outcomes.

The *K*-order Wei-Lachin test is provided by

$$\begin{array}{c} Z_{S}=\frac{J^{\prime }\hat{\beta }}{\sqrt{J^{\prime }\hat{\Sigma }J}}=\frac{J^{\prime }\hat{\beta }/K}{\sqrt{J^{\prime }\hat{\Sigma }J}/K}=\frac{\hat{\bar{\beta }}}{\sqrt{V(\hat{\bar{\beta }})}} \end{array}$$ (10)

where ***J*** = (1…1)^*T*^. Asymptotically *Z*_*S*_ ∼ *N*(0, 1) under *H*_0_ and the test rejects *H*_0_ in favor of ${\tilde{H}}_{1}=H_{1<}$ in (9) when *Z*_*S*_ ≤ *Z*_*α*_ at level *α* one-sided, or using |*Z*_*S*_| ≥ *Z*_1−*α*/2_ at level *α* two-sided. Frick [10, 11] showed that this test is maximin efficient provided that $J^{\prime }\hat{\Sigma }>0$ which will almost always apply. Then, the joint null hypothesis in (7) can be replaced by the surrogate hypothesis ${\tilde{H}}_{0}:\bar{\beta }=0$, thus satisfying the conditions in (5).

For an intermediate order test the unit vector ***J*** is modified to only include a 1 for those components tested, 0 otherwise. For example, if *K* = 4 and we wish to test the 2-order hypothesis *H*_0,24_, the test would employ the corresponding vector ***J***_24_ = (0 1 0 1)^*T*^ in the like expressions

$$\begin{array}{c} Z_{S,24}=\frac{J_{24}^{\prime }\hat{\beta }}{\sqrt{J_{24}^{\prime }\hat{\Sigma }J_{24}}}=\frac{{\hat{\bar{\beta }}}_{24}}{\sqrt{V({\hat{\bar{\beta }}}_{24})}} \end{array}$$ (11)

where ${\hat{\bar{\beta }}}_{24}$ is the mean of the coefficients tested. Then let ***D***_24_ = *diag* (***J***_24_). The corresponding maximin condition is $J_{24}^{\prime }\left(D_{24}^{\prime }\hat{\Sigma }D_{24}\right)>0$ for those elements with a corresponding value 1 in ***J***_24_.

Then the elemental hypothesis for the first component *H*_0,1_: *β*_1_ = 0 would be rejected if the tests of ${\tilde{H}}_{0,1234}\text{:}\;{\bar{\beta }}_{1234}=0$; ${\tilde{H}}_{0,123}\text{:}\;{\bar{\beta }}_{123}=0$; ${\tilde{H}}_{0,124}\text{:}\;{\bar{\beta }}_{124}=0$; ${\tilde{H}}_{0,134}\text{:}\;{\bar{\beta }}_{134}=0$; ${\tilde{H}}_{0,12}\text{:}\;{\bar{\beta }}_{12}=0$; ${\tilde{H}}_{0,13}\text{:}\;{\bar{\beta }}_{13}=0$; ${\tilde{H}}_{0,14}\text{:}\;{\bar{\beta }}_{14}=0$; and *H*_0,1_ were all nominally significant at level *α*. A similar testing tree would apply to the other elemental hypotheses.

For illustration we use data from the Prevention of Events with Angiotensin Converting Enzyme Inhibition (PEACE) study [12] that assessed whether treatment with ACE inhibition with trandolapril (ACEi, n = 4158) versus placebo (n = 4132), when added to standard therapy, would reduce the risk of cardiovascular outcomes.

Table 2 presents the numbers of subjects (cases) with each type of event, the hazard ratio, the two-sided confidence limits and *p*-value, nominally, with no adjustment for multiple tests. There is a slight benefit with ACEi versus placebo for CV death, but none for non-fatal MI. However, there is a barely non-significant (two-sided) benefit with ACEi for non-fatal stroke, and a barely significant benefit for congestive heart failure. This pattern of differences between groups represents the type of results that would fall under the one-directional alternative hypothesis (9).

**Table 2:** Numbers of subjects (cases) with each type of cardiovascular event, the ACEI versus placebo HR, 95% confidence interval and nominal two-sided p-value, not adjusted for multiple tests.

	# Cases				
	ACEi	Placebo	A:P	Nominal	Nominal
Outcome	(n = 4158)	(n = 4132)	HR	95% CI	*p*
CV death	146	152	0.951	0.758, 1.194	0.667
Non-fatal MI	222	220	1.000	0.830, 1.205	1.0
Non-fatal stroke	55	75	0.724	0.511, 1.026	0.070
CHF	105	134	0.773	0.599, 0.998	0.049

The traditional closed testing procedure would start with a *T*^2^-like omnibus *K*-order test as in (8). For the set of 4 PEACE study outcomes, this yields $X_{O}^{2}=7.39$ on 4 *df* with *p* = 0.117 and no difference between groups can be declared to reach significance.

Table 3 then presents the surrogate closed testing (two-sided) using the Wei-Lachin test for orders 2 through 4. Test results that do not reach significance at the 0.05 level, or are included in an interaction hypothesis that is not rejected, e.g. *H*_0,12_, are not shown. The order 4 initial test is significant at *p* ≤ 0.05. Of the four order 3 hypotheses, ${\tilde{H}}_{0,123}$ and ${\tilde{H}}_{0,124}$ are not significant. Since these two hypotheses include intersections of all four elementary hypotheses, then no elemental hypotheses can be rejected, i.e. all are considered non-significant.

**Table 3:** The sequence of tested hypotheses for the components of the MACE + CHF outcomes for the ACEI versus placebo groups with the mean HR, two-sided 95% confidence limits and two-sided p-value from the Wald test of the group difference in a Cox PH model. All other tests not shown are not significant at the 0.05 level. All surrogate hypotheses $\tilde{H}$ are tested using the Wei-Lachin test.

		A:P		two-sided
Order	Hypothesis (NF = non-fatal)	HR	95% CI	*p* =
4	${\tilde{H}}_{0,1234}$: CV death, NF MI, stroke and CHF	0.854	0.740, 0.986	0.032
3	${\tilde{H}}_{0,123}$: CV death, NF MI and NF stroke	0.911	0.810, 1.024	0.118
	${\tilde{H}}_{0,124}$: CV death, NF MI and CHF	0.926	0.829, 1.035	0.174
	${\tilde{H}}_{0,134}$: CV death, NF stroke and CHF	0.854	0.751, 0.972	0.017
	${\tilde{H}}_{0,234}$: NF MI, stroke and CHF	0.865	0.763, 0.981	0.024
2	${\tilde{H}}_{0,34}$: NF stroke and CHF	0.865	0.774, 0.966	0.011
1	*H*_0,3_: NF stroke	0.923	0.846, 1.006	0.070
	*H*_0,4_: NF CHF	0.938	0.880, 0.999	0.049

However, hypotheses ${\tilde{H}}_{0,134}$ and ${\tilde{H}}_{0,234}$ are each significant at *p* = 0.017 and 0.024 respectively. These are the two order-3 hypotheses that include intersections with ${\tilde{H}}_{0,34}$. This hypothesis can then be tested and indeed is significant at *p* = 0.011, indicating a treatment group difference in the joint (bivariate) event-time distributions of non-fatal stroke and CHF. Thus, by surrogate closed testing we can conclude that ACEi significantly reduced the risk of non-fatal stroke and CHF jointly, but are not able to demonstrate a beneficial effect on either outcome separately. In addition, neither would be significant had the Holm or Hochberg procedure been applied to the set of 4 component tests.

The most common method of analysis of such a composite outcome is a simple 1 *df* test of the difference between the treatment versus control groups using a logrank or Cox PH model test of the time to the first event (TTFE). This could also be viewed as providing a test of a different surrogate hypothesis that the distribution of the minimum event time does not differ between groups. This approach, however, does not include other events following the initial event, such as a CV death that occurs after an initial non-fatal MI. Lachin and Bebu [7] also show that the Wei-Lachin test can be more powerful than the TTFE analysis.

For the PEACE study, the analysis of the MACE + CHF composite outcome using the TTFE yields an estimated hazard ratio of 0.90 with a 95% confidence interval of (0.79, 1.02) with *p* = 0.12 two-sided. Thus, closed testing of the PEACE outcomes using either the omnibus or the TTFE test fails to declare any significant difference between groups.

Further, a note of caution. Bebu and Lachin [13] also show that the TTFE may not provide an unbiased *α*-level test of the joint null hypothesis that the hazard or survival functions do not differ between groups, i.e. of *H*_0_: ***β*** = 0. Let $\tilde{\beta }$ denote the log (HR) for the time-to-first event. They show that the distribution of the estimate $\hat{\tilde{\beta }}$ can differ substantially among groups even when *H*_0_ in (7) is true, and conversely that there may be no difference between groups in the distribution of $\hat{\tilde{\beta }}$ even though *H*_0_ is false. These discrepancies occur when there is a difference between groups in the correlation structure of the component event times. Unfortunately, there is no general method to assess this difference in correlations; however, Bebu and Lachin [13] describe an estimate of the correlation of event times under a bivariate exponential distribution.

### Longitudinal repeated measures

Consider the case of *K* repeated measures over time where it is desired to conduct a test of the difference between the group means at each of the *K* points in time, post-randomization. Let *μ*_*ij*_ denote the mean of the observations in the *i*th group at the *j*th time, and *θ*_*j*_ = *μ*_1*j*_ − *μ*_2*j*_ denote the mean difference at the *j*th time. The *K* differences could be tested using a Bonferroni-type procedure, such as that of Holm. Alternately, a traditional closed testing procedure could be conducted starting with an overall omnibus *K* *df* “MANOVA” test using a *T*^2^-test, with successive sub-order *T*^2^ tests.

However, another possible order-*K* test is the overall group effect on 1 *df* in a longitudinal model that compares the “LSMEANS” of the two groups, these being the model-estimated average of the means over time in the two groups. Again, consider the case of *K* = 4 where ${\hat{\theta }}_{J}={\hat{\mu }}_{1J}-{\hat{\mu }}_{2J}$ and the ${\hat{\mu }}_{iJ}$ in the *i*th group at the *j*th time are obtained from a repeated measures longitudinal model. Then the estimated LSMEAN of the 4 repeated measures combined in the *i*th group is the unweighted mean ${\hat{\bar{\mu }}}_{i,1234}$ and the estimated LSMEAN difference is ${\hat{\bar{\theta }}}_{1234}={\hat{\bar{\mu }}}_{1,1234}-{\hat{\bar{\mu }}}_{2,1234}$. Thus, at order *K*, the 1 *df* test of the difference in the LSMEANS of the *K* repeated measures is employed that provides a test of the surrogate hypothesis ${\tilde{H}}_{0,1234}:{\bar{\theta }}_{1234}=0$. At order *K* − 1, the LSMEANS of a given set of *K* − 1 means is employed, such as a test of ${\tilde{H}}_{0,123}:{\bar{\theta }}_{123}=0,$ and so on. Then at order 1 the difference between groups in the means at the *j*th time could be tested using a simple *t*-test provided that all of the intersection hypotheses of LSMEANS containing the *j*th mean difference are significant at level *α*. This approach would be directed to alternative hypotheses where the mean differences over time were all in the same direction, i.e. the mean profiles did not cross, analogous to the alternative hypothesis in (9).

For example, an analysis of the group differences in *K* = 4 repeated measures can be conducted using SAS PROC MIXED with a nested model using statements such as

PROC MIXED METHOD = ML;

 class id time group;

 model = X time group(time);

 repeated / type = un subject = id;

 lsmeans group(time) / pdiff cov;

where *X* is the baseline value, *time* is a class variable with 4 levels and *group* is a class variable with 2 levels. The *group(time)* estimated coefficients are the differences in the group means at each time. Then an estimate of the difference between the group LSMEANS over the *K* points in time $\left({\hat{\bar{\theta }}}_{1234}\right)$ is obtained using an estimate statement such as

estimate ‘4Level’ group(time) 0.25 -0.25 0.25 -0.25 0.25 -0.25

0.25 -0.25;

that also provides a 1 *df* test of the group difference in LSMEANS. Then, for example, a test of the group differences at times 1, 2 and 4, and the estimate of the average group difference over these times $\left({\hat{\bar{\theta }}}_{124}\right)$, would be provided by a statement such as

estimate '3Level 1.2.4' group(time) 0.3333 -0.3333 0.3333 -0.3333 0 0

0.3333 -0.3333;

A set of such statements can then provide tests of all the intersection hypotheses for the *K* repeated measures.

Also note that since the test of the LSMEANS is a test of the unweighted average of the time-specific means, then this is the same as a Wei-Lachin one-directional test. Lachin [14] also describes the details of the application of the Wei-Lachin test to multiple mean differences. This test is efficient when the groups tend to differ in the same direction, but not necessarily of the same magnitude, over time.

To illustrate, consider an analysis of the systolic blood pressure values recorded every 6 months over the first 2 years of follow-up in the subset of 1371 subjects with diabetes in the PEACE study. Had the full cohort of 8290 subjects been employed, virtually every method of analysis would produce extremely significant differences. The following are the treatment group within time LSMEANS and the LSMEAN differences (placebo—ACEi):

**Table:** 

	LSMEAN				
Month	ACEi	Placebo	Di.	S.E.	*p*-value
6	131.8	136.0	4.2	0.87	<0.0001
12	131.7	135.1	3.4	0.93	0.0002
18	131.7	134.0	2.3	0.93	0.0134
24	132.5	134.5	2.2	0.97	0.0383

Table 4 then shows that all tests of the higher order intersection hypotheses are significant at the 0.05 level so that the elementary hypotheses can also be tested at the 0.05 level and all are significant.

**Table 4:** The sequence of tested hypotheses for the longitudinal analysis of systolic blood pressure in the subset of diabetic subjects in the PEACE study. The model is adjusted for the baseline systolic blood pressure and the group differences tested using a *t*-test with 1288 *df*. Shown is the tested hypothesis for each intersection hypothesis, ($\bar{\theta }$), the difference in the LSMEANS for placebo minus ACEi, the SE and the two-sided *p*-value for the test of the difference between groups. For example, the test of ${\bar{\theta }}_{124}$ is testing that the average of the group means at visits 1, 2 and 4 (6, 12 and 24 months) is the same in the two groups.

	Diff.		two-sided
Hypothesis	P-A	*SE*	*p*-value
${\bar{\theta }}_{1234}=0$	2.9668	0.6444	<0.0001
${\bar{\theta }}_{123}=0$	3.2881	0.6755	<0.0001
${\bar{\theta }}_{124}=0$	3.1895	0.6659	<0.0001
${\bar{\theta }}_{134}=0$	2.8178	0.6796	<0.0001
${\bar{\theta }}_{234}=0$	2.5708	0.7014	0.0003
${\bar{\theta }}_{12}=0$	3.7837	0.7255	<0.0001
${\bar{\theta }}_{13}=0$	3.2261	0.7372	<0.0001
${\bar{\theta }}_{14}=0$	3.0782	0.7218	<0.0001
${\bar{\theta }}_{23}=0$	2.8555	0.7574	0.0002
${\bar{\theta }}_{24}=0$	2.7076	0.7647	0.0004
${\bar{\theta }}_{34}=0$	2.1499	0.7816	0.0060

In comparison, had the 4 elementary hypotheses been tested using the Holm procedure, all would also have been significant at the 0.05 level, the adjusted *p*-values for months 6, 12, 18 and 24 (ranked in that order) are <0.0004, 0.0006, 0.0268 and 0.0383.

## Subgroup analyses

### Closed testing of group differences within subgroups

Consider the case where pre-specified analyses of the differences between groups are conducted within *K* = 2 subgroups of the study population defined by a subgroup factor, such as the comparison of treatment group differences separately among men and among women (later generalized to *K* ≥ 2 subgroups). It is generally recommended that analyses within subgroups only be conducted when a test for a group by subgroup factor interaction, or a test for homogeneity of effects among subgroups, is significant [15], such as a test that the treatment group difference among males equals that among females. If significant, then the tests of significance within each subgroup often employ an alpha adjustment for the 2 tests, such as a Bonferroni correction (or its generalizations). However, a correction is unnecessary under the surrogacy principle described above.

Let {*θ*_*j*_} denote the treatment group difference within the *j* th subgroup, *j* = 1,2, defined by the gender of each subject, where *θ*_1_ is the treatment group difference among males and *θ*_2_ the difference among females. Then $\hat{\theta }=({\hat{\theta }}_{1}\;{\hat{\theta }}_{2}{)}^{T}$ is asymptotically normally distributed with expectation ***θ*** = (*θ*_1_ *θ*_2_)^*T*^ and with a covariance matrix $\Sigma =diag(\sigma_{1}^{2}\;\sigma_{2}^{2})$ with covariance *σ*_12_ = 0 since the two subgroups are independent.

The objective is to determine whether the treatment group difference within either subgroup is statistically significant when there is heterogeneity of the treatment group differences among the two subgroups. Thus, the elemental null hypotheses to be tested are *H*_0,1_: *θ*_1_ = 0 and *H*_0,2_: *θ*_2_ = 0. One approach is to use a Bonferroni correction for the two tests. Another is to use traditional closed sequential testing that would start with a *T*^2^-like Wald test of the joint null hypothesis *H*_0,12_: *θ*_1_ = *θ*_2_ = 0 against the global or omnibus alternative *H*_1,12_: *θ*_1_ ≠ 0 and/or *θ*_2_ ≠ 0 of a group difference in either direction within either subgroup. With a consistent estimate $\hat{\Sigma }$, this order 2 test is provided by

$$\begin{array}{ccc} X_{O}^{2} & = & {\hat{\theta }}^{\prime }{\hat{\Sigma }}^{-1}\hat{\theta }=Z_{1}^{2}+Z_{2}^{2}\;\text{where}\\ Z_{j} & = & {\hat{\theta }}_{j}/{\hat{\sigma }}_{j},\;j=1,2. \end{array}$$ (12)

Under *H*_0,12_, $X_{O}^{2}$ is distributed as chi-square on 2 *df*. If significant at level *α*, each of the elemental hypotheses *H*_0,1_ and *H*_0,2_ are rejected if the corresponding *Z*-test values are likewise significant at level *α*.

However, the alternative hypothesis parameter space (*H*_1,12_) for this order 2 test includes cases where *θ*_1_ = *θ*_2_ ≠ 0, i.e. where there is a homogeneous non-zero treatment group difference within the two subgroups. Such values do not represent any heterogeneity among subgroups or a treatment by subgroup interaction. Thus, the order 2 omnibus test is not specifically directed to detecting cases where there is a treatment by subgroup interaction.

Rather, we only wish to assess the treatment effect within subgroups when there is evidence that the variation among subgroups is greater than would be expected by chance, i.e. a treatment by subgroup interaction exists. So in this case we are interested in first testing the surrogate null hypothesis ${\tilde{H}}_{0,12}:\theta_{1}=\theta_{2}$ against ${\tilde{H}}_{1,12}:\theta_{1}\neq \theta_{2}$. A simple test is provided by

$$\begin{array}{c} Z_{S}=\frac{{\hat{\theta }}_{1}-{\hat{\theta }}_{2}}{\sqrt{{\hat{\sigma }}_{1}^{2}+{\hat{\sigma }}_{2}^{2}}}. \end{array}$$ (13)

Asymptotically *Z*_*S*_ ∼ *N*(0, 1) under ${\tilde{H}}_{0,12}$ and the test rejects ${\tilde{H}}_{0,12}$ in favor of ${\tilde{H}}_{1,12}$ when *Z*_*S*_ ≥ *Z*_1−*α*_ for an upper-tail one-sided test at level *α*, or when *abs*(*Z*_*S*_) ≥ *Z*_1−*α*/2_ at level *α* two-sided. If that test is significant, we can then test the treatment difference within each subgroup at level *α* (two-sided) with strong control of the type 1 error probability, without the need for a correction for two tests.

Again, note that $H_{0,12}\Rightarrow {\tilde{H}}_{0,12}$ and rejection of ${\tilde{H}}_{0,12}\Rightarrow$ rejection of *H*_0,12_. In this case, the order 2 joint hypothesis (*H*_0,12_) of no difference in both subgroups implies that both subgroups have the same null effect $\left({\tilde{H}}_{0,12}\right)$. However, if we reject ${\tilde{H}}_{0}$ this implies that the no-interaction hypothesis *H*_0,12_ is false because *θ*_1_ ≠ *θ*_2_ implies that *θ*_1_ and *θ*_2_ cannot both equal zero.

This can also be generalized to the case of more than 2 subgroups. Suppose *K* = 3 with the vector of estimated treatment group differences within the three subgroups $\hat{\theta }={\left[{\hat{\theta }}_{1}\;{\hat{\theta }}_{2}\;{\hat{\theta }}_{3}\right]}^{T}$. Since the subgroups are independent, the covariance matrix of the treatment group estimates within the three subgroups is $\hat{\Sigma }=diag[{\hat{\sigma }}_{1}^{2}\;{\hat{\sigma }}_{2}^{2}\;{\hat{\sigma }}_{3}^{2}].$ In this case the traditional 3-order test of *H*_0,123_ would be replaced by a 2 *df* test of homogeneity of the three subgroups differences ${\tilde{H}}_{0,123}:\theta_{1}=\theta_{2}=\theta_{3}$ using a *T*^2^ -like statistic of the form in (2) with contrast matrix

$$\begin{array}{c} C^{\prime }=[\begin{array}{ccc} 1 & -1 & 0\\ 1 & 0 & -1 \end{array}] \end{array}$$ (14)

with subgroup 1 as the reference for the 2:1 and 3:1 pairwise subgroup differences. Then the test of the elemental hypothesis *H*_0,1_, for example, would be declared significant at level *α* if it and the intersection hypotheses ${\tilde{H}}_{0,12}$, ${\tilde{H}}_{0,13}$, and ${\tilde{H}}_{0,123}$ were all rejected at level *α*. The other elemental hypotheses can likewise be tested at level *α* provided that the relevant higher order intersection hypotheses are also rejected at level *α*.

### Numerical computations

Computations were conducted for the case of two (independent) subgroups to compare the operating characteristics of the traditional closed testing approach for subgroup analyses versus analyses using the test of the surrogate hypothesis of homogeneity. Computations also included tests within 2 subgroups using a Holm (improved Bonferroni) correction that were virtually identical to the traditional closed testing and are omitted herein. To simplify, we assume that the variance of the observations is 1 with sample size *n* per treatment group in both subgroups so that the standard error of the mean difference within each subgroup is ${\hat{\sigma }}_{j}=\sqrt{2/n}$.

The traditional closed testing approach employs a 2 *df* omnibus *T*^2^-like test of the order-2 hypothesis *H*_0,12_: *θ*_1_ = *θ*_2_ = 0 shown in (12). Under *H*_0,12_ the test statistic $X_{O}^{2}=Z_{1}^{2}+Z_{2}^{2}$ has a large-sample central Chi-square distribution with 2 *df*. The null is rejected at the *α* = 0.05 level if the statistic is greater than the distribution’s 95th percentile. If significant, both *H*_0,1_ and *H*_0,2_ can be tested at the 0.05 level, either one or two-sided. Herein all tests are conducted two-sided at the 0.05 level.

Alternately, at order 2 we could employ the 1 *df* test of the surrogate hypothesis of homogeneity ${\tilde{H}}_{0,12}:\theta_{1}=\theta_{2}$. Under ${\tilde{H}}_{0,12}$, the contrast test statistic $Z_{S}=\left({\hat{\theta }}_{1}-{\hat{\theta }}_{2}\right)/\sqrt{2/n}$ from (13) has a large-sample standard normal distribution. If this test of homogeneity is significant at level *α* two-sided, then both *H*_0,1_ and *H*_0,2_ can be tested at level *α* = 0.05 one or two-sided.

**Fig 1:**
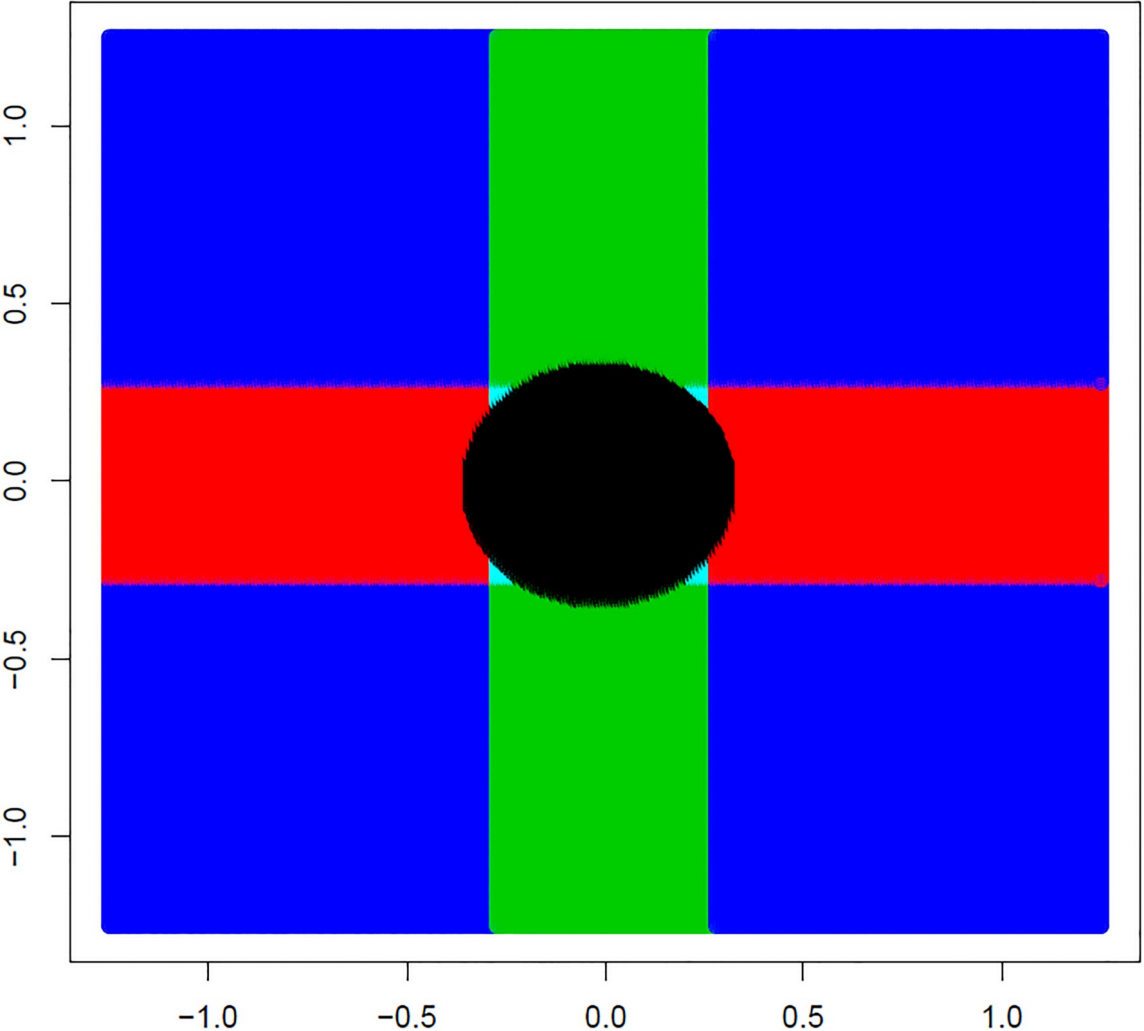
Rejection regions for traditional closed testing. Plot of rejection regions for tests under the traditional closed testing procedure. The omnibus two degree-of-freedom test of *H*_0,12_: *θ*_1_ = *θ*_2_ = 0 will reject the null hypothesis at level *α* for values (${\hat{\theta }}_{1},{\hat{\theta }}_{2}$) outside the circle. If the omnibus test is significant at level *α*, the test of *H*_0,1_: *θ*_1_ = 0 then rejects outside of the green bar, and that of *H*_0,2_: *θ*_2_ = 0 rejects outside of the red bar. Note the four small near-triangles in which the omnibus test is rejected but neither test of the two elementary tests is significant.

Figures describe the difference between the traditional and surrogate testing procedures. Fig 1 illustrates the rejection region for the traditional method starting with the 2 *df* omnibus test of *H*_0,12_: *θ*_1_ = *θ*_2_ = 0 at level *α* = 0.05, followed by 1 *df* tests of *H*_0,1_ and *H*_0,2_, two-sided. The omnibus test rejection region at *α* = 0.05 consists of points $\left({\hat{\theta }}_{1},{\hat{\theta }}_{2}\right)$ outside of the circle. If this test is significant, the hypotheses *H*_0,1_: *θ*_1_ = 0 and/or *H*_0,2_: *θ*_2_ = 0 for each subgroup may be rejected at *α* = 0.05 (two-sided) when |*Z*_*j*_| exceeds *Z*_1−*α*/2_ = *Z*_0.975_, *j* = 1, 2. For the test of *H*_0,1_ the rejection region falls outside a vertical band with a small crescent piece removed from the left and right sections. These represent values that fail to reject the joint hypothesis for which *H*_0,1_ is not tested. Likewise, the rejection region for the test of *H*_02_ falls outside a horizontal band with a small crescent removed from the upper and lower sections. Also note that there are 4 small triangular areas that fall within the rejection region for the joint test but for which the test of *H*_0,1_ or *H*_0,2_ would not be significant.

**Fig 2:**
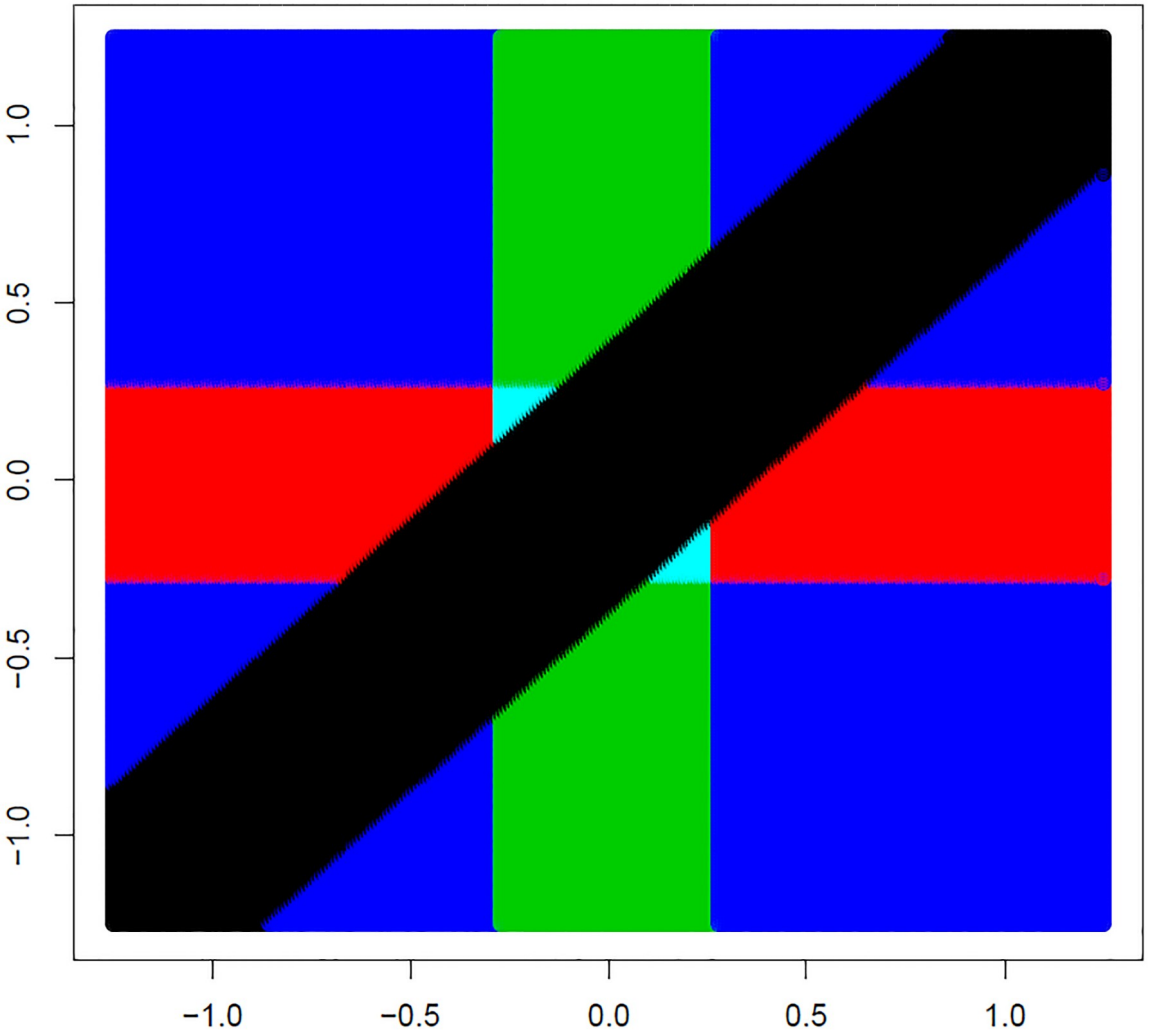
Rejection regions for surrogate closed testing. Plot of rejection region for tests under the surrogate closed testing procedure. The test of homogeneity ${\tilde{H}}_{0,12}:\theta_{1}=\theta_{2}$ will reject the null hypothesis at level *α* for values (${\hat{\theta }}_{1},{\hat{\theta }}_{2}$) outside of the black diagonal band. If the surrogate test is significant at level *α*, the test of *H*_0,1_: *θ*_1_ = 0 then rejects outside of the green bar, and that of *H*_0,2_: *θ*_2_ = 0 rejects outside of the red bar.

Fig 2 illustrates the rejection region for the surrogate test method starting with the 1 *df* contrast test of homogeneity of the subgroup mean differences ${\tilde{H}}_{0,12}:\theta_{1}=\theta_{2}$ at level *α* = 0.05 two-sided, followed by 1 *df* tests of the difference within each subgroup, two-sided. The 1 *df* test of homogeneity rejects null hypothesis for points $\left({\hat{\theta }}_{1},{\hat{\theta }}_{2}\right)$ outside of a diagonal band about the line of equality ${\hat{\theta }}_{1}={\hat{\theta }}_{2}$. Outside of this band the difference between ${\hat{\theta }}_{1}$ and ${\hat{\theta }}_{2}$ is large enough to reject ${\tilde{H}}_{0,12}$. Then the hypothesis *H*_0,1_ for the first subgroup mean difference is rejected at *α* = 0.05 (two-sided) when $|{\hat{\theta }}_{1}|$ exceeds *Z*_1−*α*/2_ = *Z*_0.975_. This corresponds to a vertical band symmetric about *θ*_1_ = 0. Likewise, for the test of *θ*_2_ there would be a horizontal band intersecting the diagonal band that defines the rejection region. For example, the point $\left({\hat{\theta }}_{1},{\hat{\theta }}_{2}\right)=\left(5,1\right)$ falls outside of the diagonal band and therefore would indicate rejection of the test of homogeneity (rejection of ${\tilde{H}}_{0,12}:\theta_{1}=\theta_{2}$). Then the test of significance of *H*_0,1_: *θ*_1_ = 0 would be declared significant but not the test of *H*_0,2_: *θ*_2_ = 0. Also, the two small triangular areas represent values that would lead to rejection of the surrogate hypothesis of homogeneity but for which neither test within subgroups would be significant.

Table 5 then presents the operating characteristics (rejection probabilities) for tests using traditional closed-testing and surrogate closed-testing for illustrative values of *θ*_1_ and *θ*_2_ with sample sizes of *n* = 25 or 50 within each cell. These were computed using numerical integration, see the Appendix. Scenarios include values of *θ*_1_ and *θ*_2_ satisfying *H*_0,12_ and/or ${\tilde{H}}_{0,12}$ and the respective alternatives.

**Table 5:** Probabilities of rejection of the Order-2 gate-keeping tests and the tests of the elemental hypotheses using the traditional closed-testing procedure and the extended surrogate closed-testing procedure for *n* of 25 or 50 per group within each subgroup and with homogeneous or heterogeneous treatment effects *θ*_1_ and *θ*_2_ within each of the two subgroups. All tests at the 0.05 level two-sided.

			Closed			Surrogate		
	*θ*_1_	*θ*_2_	*H*_0,12_	*H*_01_	*H*_02_	${\tilde{H}}_{0,12}$	*H*_01_	*H*_02_
*n* = 25								
1	0.0	0.0	0.0500	0.0249	0.0249	0.0500	0.0169	0.0169
2	0.5	0.5	0.6027	0.3825	0.3825	0.0500	0.0247	0.0247
3	1.0	1.0	0.9965	0.9419	0.9419	0.0500	0.0366	0.0366
4	0.0	0.5	0.3335	0.0408	0.3069	0.2394	0.0230	0.1964
5	0.0	1.0	0.8962	0.0497	0.8921	0.7054	0.0267	0.6983
6	0.5	1.0	0.9523	0.4224	0.9254	0.2394	0.0189	0.2387
*n* = 50								
1	0.0	0.0	0.0500	0.0249	0.0249	0.0500	0.0169	0.0169
2	0.5	0.5	0.8962	0.6917	0.6917	0.0500	0.0267	0.0267
3	1.0	1.0	1.0000	0.9988	0.9988	0.0500	0.0492	0.0492
4	0.0	0.5	0.6028	0.0469	0.5857	0.4240	0.0247	0.3954
5	0.0	1.0	0.9965	0.0500	0.9964	0.9425	0.0366	0.9423
6	0.5	1.0	0.9995	0.7056	0.9986	0.4240	0.1921	0.4239

For each sample size, under the joint null hypothesis *H*_0,12_: *θ*_1_ = *θ*_2_ = 0 in scenario 1, all tests have a type I error probability ≤ 0.05, with that for the surrogate tests within each subgroup being less (more conservative) than traditional closed testing. Under the surrogate joint null hypothesis ${\tilde{H}}_{0,12}:\theta_{1}=\theta_{2}=0.5$ or 1.0 (scenarios 2-3), the rejection probabilities for the surrogate tests of the elementary hypotheses, the type I error probability for these tests, is ≤ 0.05. However, scenarios 2 and 3 also fall under the global alternative *H*_1,12_ for which, as would be expected, the traditional closed testing procedures provide increasing power as the common value for *θ* increases. This is also reflected by the power of the 2 *df* test of *H*_0,12_ under the joint null compared to the nominal type I error probabilities of the 1 *df* test of the surrogate hypothesis ${\tilde{H}}_{0,12}$.

Scenarios 4-6 fall under both the global alternative hypothesis *H*_1,12_ and the surrogate alternative hypothesis ${\tilde{H}}_{1,12}$ where 0 ≤ *θ*_1_ < *θ*_2_. In scenarios 4 and 5 where *θ*_1_ = 0, all procedures preserve the type I error probability for the test of *H*_0,1_ and the traditional closed testing procedure provides slightly greater power for the test of *H*_02_ than does the surrogate test (∼0.996 versus 0.942 when *θ*_2_ = 1.0 for *n* = 50). However, in scenario 6 where *θ*_1_ = 0.5 and *θ*_2_ = 1.0, since the difference between subgroups is smaller than scenario 5 (0.5 versus 1.0), the surrogate test of ${\tilde{H}}_{0,12}$ is less powerful than the traditional omnibus test of *H*_0,12_ (0.424 versus nearly 1.0 for *n* = 50), and as a result, the tests of the elementary hypotheses are less powerful under the surrogate versus traditional closed testing.

Note that scenarios 2-3 fall under the global alternative *H*_1,12_ whereas they fall under the surrogate null hypothesis ${\tilde{H}}_{0,12}$. Thus, the traditional tests have greater “power”. Scenarios 4-6 fall under both alternatives. In all cases the traditional closed tests have higher rejection probabilities. That is because they are rejecting *H*_0,12_ in situations that do not fall in the surrogate alternative ${\tilde{H}}_{1,12}$ parameter space.

To show this consider the following 2×2 table for scenario 4 and *n* = 50 that displays the joint and marginal probabilities that the elementary test within stratum 2 would be significant at the 0.05 level using either the traditional or the surrogate closed testing procedures.

$$\begin{array}{cc} & Surrogate\;Test\;\\ \begin{array}{c} Traditional\;Test \end{array} & \begin{array}{ccc} & \begin{array}{cccc} + & & & - \end{array} & \\ \begin{array}{c} +\\ - \end{array} & \begin{array}{cc} 0.374 & 0.212\\ 0.212 & 0.393 \end{array} & \begin{array}{c} 0.586\\ 0.414 \end{array}\\ & \begin{array}{cc} 0.395 & 0.605 \end{array} & 1.0 \end{array} \end{array}$$ (15)

Marginally, the traditional closed testing procedure has a higher rejection probability than does the surrogate closed testing (0.586 versus 0.395). However, the probability that both reject is 0.374 meaning that the probability is 0.212 that the traditional test would reject in cases where the surrogate test does not, or in cases where the test of homogeneity is not significant. Further, significance of the surrogate test (with probability 0.395) is highly concordant with that of the traditional test (probability 0.374), meaning that the probability of the traditional test failing to be significant when the surrogate test is significant is small (0.021).

In summary, all procedures preserve the type I error probability under the null for either or both elementary tests (scenarios 1-3). Under the surrogate alternative ${\tilde{H}}_{1,12}$ (scenarios 4-6), the traditional testing procedure provides greater “power” than the surrogate testing owing to a higher probability of rejection in cases where ${\tilde{H}}_{0,12}$ is true, i.e. the treatment group differences are dissimilar. Thus, the rejection regions for the traditional versus surrogate closed testing procedures differ, as well as the probabilities of rejection over the parameter space.

**Fig 3:**
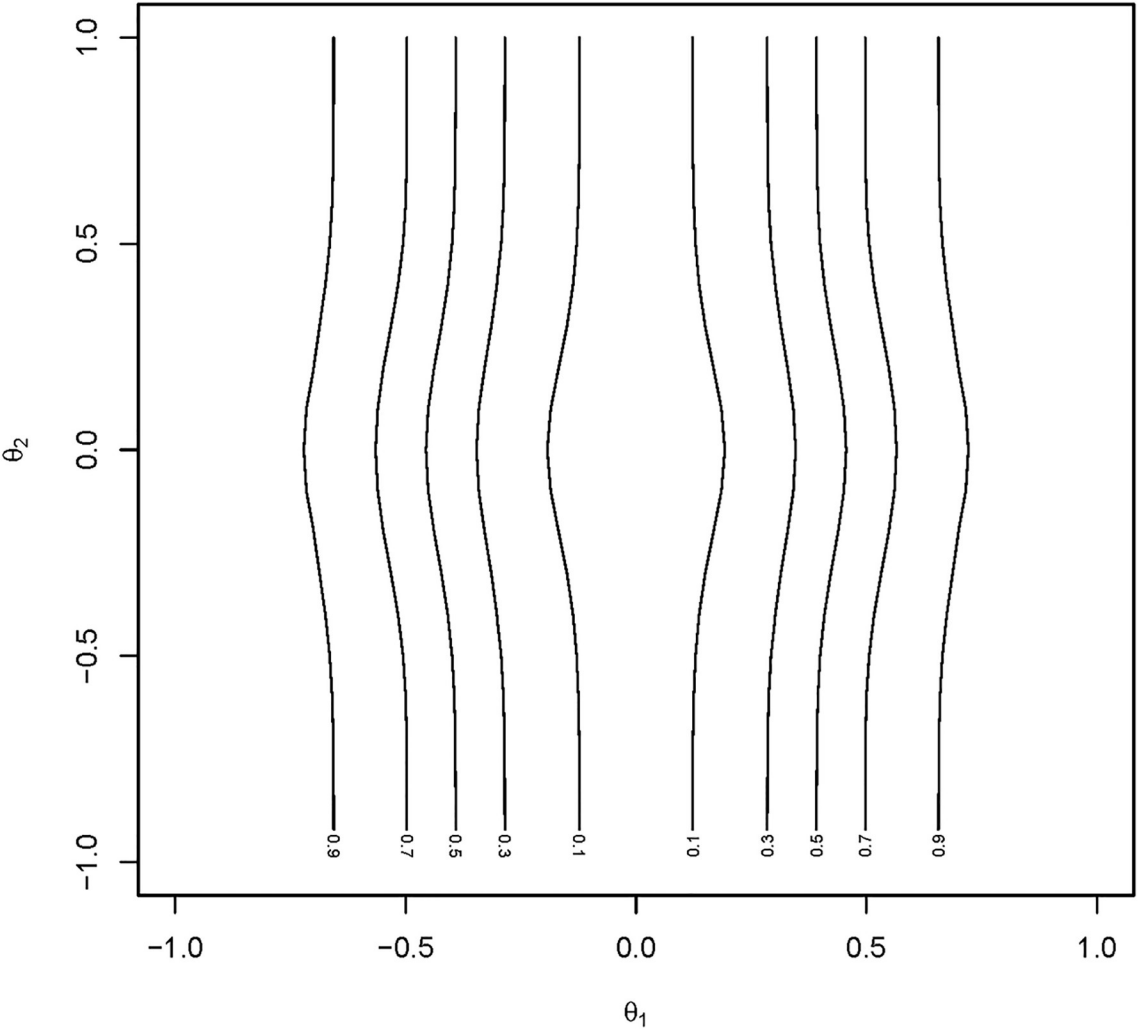
Power contours for traditional closed testing. Plot of power contours for the test of the elemental hypothesis *H*_0,1_ under the traditional closed testing procedure. Power contours for the test of *H*_0,2_ are identical when *θ*_2_ is interchanged with *θ*_1_.

To display this, the probability of rejection of the different tests was computed by numerical integration for *θ*_1_ = −1(0.1)1 and *θ*_2_ = −1(0.1)1. The values of *θ*_1_ and *θ*_2_ for which power equaled a specific value were then plotted (power contours). Fig 3 displays the power contours over the parameter space for the tests of the elementary hypotheses *H*_0,1_ and *H*_0,2_, respectively, for the traditional closed testing procedure for *n* = 50. These power contours are close to straight vertical or horizontal lines, respectively, as would be the case for a simple test with no adjustment for multiplicity.

**Fig 4:**
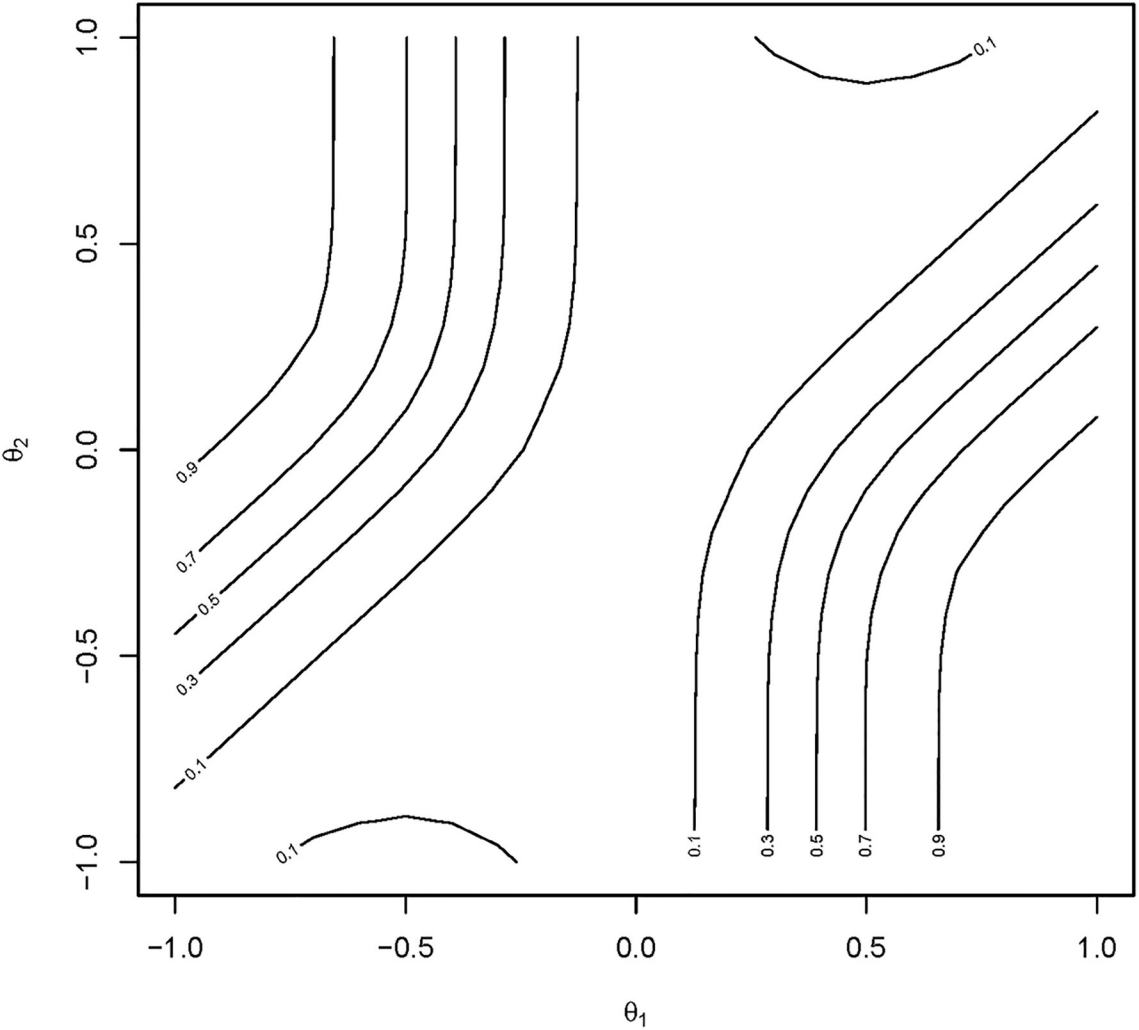
Power contours for surrogate closed testing. Plot of power contours for the test of the elemental hypothesis *H*_0,1_ under the surrogate closed testing procedure. Power contours for the test of *H*_0,2_ are identical when *θ*_2_ is interchanged with *θ*_1_.

Fig 4 then displays the power contours for these same tests using the surrogate closed testing procedure. The regions in which the test of *H*_0,1_ has high power, such as 0.7 or greater, are characterized by vertical lines in the upper left and lower right quadrants that “bend” away from the diagonal acceptance region for the surrogate test of ${\tilde{H}}_{0,12}$. The same pattern is obtained for the test of *H*_0,2_ when the labels of the axes are interchanged. Thus, these contours describe regions of the parameter space where the *θ*_1_ and *θ*_2_ within the two subgroups differ substantially, and where there is a high probability that a test of either *θ*_1_ and/or *θ*_2_ would also be significant.

## Discussion

Herein we describe applications of the closed testing principle using *α*-level tests of higher order surrogate hypotheses that are directed to testing different null versus alternative hypotheses than those employed in traditional closed-testing procedures. The type I error probability is protected provided that all hypotheses are tested using an *α*-level test. We present three applications directly relevant to the analysis of clinical trial results. Clearly there are others. The advantage of the surrogate testing approach is that it provides a test that is directed to detect specific alternatives of interest, as opposed to the global alternative hypothesis of any difference in any direction.

The first two examples both employ surrogate hypotheses that are directed towards regions of the parameter space where one group has a preponderance of benefit for the set of outcomes considered, the so-called one-directional alternative hypothesis (9). This alternative is specified in terms of one group being more beneficial than the other, such as the experimental treatment being beneficial relative to placebo. However, there may be situations, such as a study of comparative effectiveness, where it is of interest to determine whether either treatment A is superior to B or vice versa, in which case a two-sided alternative hypothesis and two-sided test would be employed. A two-sided analysis can also be employed to meet regulatory requirements to establish effectiveness in a placebo controlled trial.

## Appendix

Since the statistics ${\hat{\theta }}_{1}$ and ${\hat{\theta }}_{2}$ are independent then the joint density of *Z*_1_ and *Z*_2_ is the product of two normal densities *ϕ*_*j*_(*z*_*j*_) with means $\mu_{j}=\theta_{j}\sqrt{n/2}$, *j* = 1, 2, and variances 1.0. Then for given values (*θ*_1_, *θ*_2_) the expected value of some function of *Z*_1_ and *Z*_2_, say *g*(*z*_1_, *z*_2_), was computed numerically as

$$\begin{array}{c} E[g(z_{1},z_{2})]=\int_{\mu_{1}-5}^{\mu_{1}+5}\int_{\mu_{2}-5}^{\mu_{2}+5}g\left(z_{1},z_{2}\right)\;\phi_{1}\left(z_{1}\right)\;\phi_{2}\left(z_{2}\right)\;d\left(z_{1}\right)\;d\left(z_{2}\right) \end{array}$$

where *d*(*z*_1_) = *d*(*z*_2_) = 0.001. The functions herein are simple indicator functions with expectations being the probabilities of significance of specific tests of interest, such as

**Table:** 

Test	*g*(*z*_1_, *z*_2_)
2 *df* Omnibus	$I(X_{O}^{2}\geq X_{2,1-\alpha }^{2})$
Closed *Z*_1_	$I\left(X_{O}^{2}\geq X_{2,1-\alpha }^{2}\right)I\left(Z_{1}\geq Z_{1-\alpha }\right)$
Closed *Z*_2_	$I\left(X_{O}^{2}\geq X_{2,1-\alpha }^{2}\right)I\left(Z_{2}\geq Z_{1-\alpha }\right)$
Homogeneity (*Z*_*S*_)	*I*(*Z*_*S*_ ≥ *Z*_1−*α*_)
Surrogate Closed *Z*_1_	*I*(*Z*_*S*_ ≥ *Z*_1−*α*_)*I*(*Z*_1_ ≥ *Z*_1−*α*_)
Surrogate Closed *Z*_2_	*I*(*Z*_*S*_ ≥ *Z*_1−*α*_)*I*(*Z*_2_ ≥ *Z*_1−*α*_)
Holm *Z*_1_	*I*(*Z*_1_ ≥ *Z*_2_)*I*(*Z*_1_ ≥ *Z*_1−*α*/2_)+
	*I*(*Z*_2_ ≥ *Z*_1_)*I*(*Z*_2_ ≥ *Z*_1−*α*/2_)*I*(*Z*_1_ ≥ *Z*_1−*α*_)
Holm *Z*_2_	*I*(*Z*_2_ ≥ *Z*_1_)*I*(*Z*_2_ ≥ *Z*_1−*α*/2_)+
	*I*(*Z*_1_ ≥ *Z*_2_)*I*(*Z*_1_ ≥ *Z*_1−*α*/2_)*I*(*Z*_2_ ≥ *Z*_1−*α*_)

## References

[1] Marcus R, Eric P, Gabriel KR. On closed testing procedures with special reference to ordered analysis of variance. Biometrika. 1976;63(3):655–660. 10.1093/biomet/63.3.655

[2] Hsu J. Multiple comparisons: theory and methods. Chapman and Hall/CRC; 1996.

[3] Henning KS, Westfall PH. Closed testing in pharmaceutical research: Historical and recent developments. Statistics in biopharmaceutical research. 2015;7(2):126–147. 10.1080/19466315.2015.1004270 26366251 PMC4564263

[4] Lehmacher W, Wassmer G, Reitmeir P. Procedures for two-sample comparisons with multiple endpoints controlling the experimentwise error rate. Biometrics. 1991;47(2):511–521. 10.2307/2532142 1912258

[5] O’Brien PC. Procedures for comparing samples with multiple endpoints. Biometrics. 1984; p. 1079–1087. 10.2307/2531158 6534410

[6] Wassmer G, Reitmeir P, Kieser M, Lehmacher W. Procedures for testing multiple endpoints in clinical trials: an overview. Journal of statistical planning and inference. 1999;82(1-2):69–81. 10.1016/S0378-3758(99)00032-4

[7] Lachin JM, Bebu I. Application of the Wei–Lachin multivariate one-directional test to multiple event-time outcomes. Clinical Trials. 2015;12(6):627–633. 10.1177/1740774515601027 26336199 PMC4562325

[8] Wei LJ, Lin DY, Weissfeld L. Regression analysis of multivariate incomplete failure time data by modeling marginal distributions. Journal of the American statistical association. 1989;84(408):1065–1073. 10.1080/01621459.1989.10478873

[9] Lin DY, Wei LJ. The robust inference for the Cox proportional hazards model. Journal of the American statistical Association. 1989;84(408):1074–1078. 10.1080/01621459.1989.10478874

[10] Frick H. A maxmin linear test of normal means and its application to lachin’s data. Communications in statistics-theory and methods. 1994;23(4):1021–1029. 10.1080/03610929408831302

[11] Frick H. Comparing Trials with Multiple Outcomes: The Multivariate One-Sided Hypothesis with Unknown Covariances. Biometrical journal. 1995;37(8):909–917. 10.1002/bimj.4710370803

[12] Investigators PT. Angiotensin-converting–enzyme inhibition in stable coronary artery disease. New England Journal of Medicine. 2004;351(20):2058–2068. 10.1056/NEJMoa042739 15531767 PMC2556374

[13] Bebu I, Lachin JM. Properties of composite time to first event versus joint marginal analyses of multiple outcomes. Statistics in medicine. 2018;37(27):3918–3930. 10.1002/sim.7849 29956365 PMC6615937

[14] Lachin JM. Applications of the Wei-Lachin multivariate one-sided test for multiple outcomes on possibly different scales. PloS one. 2014;9(10):e108784 10.1371/journal.pone.0108784 25329662 PMC4201485

[15] Wang R, Lagakos SW, Ware JH, Hunter DJ, Drazen JM. Statistics in medicine reporting of subgroup analyses in clinical trials. New England Journal of Medicine. 2007;357(21):2189–2194. 10.1056/NEJMsr077003 18032770

